# Statistical learning of anomalous regions in complex faux X-ray images does not transfer between detection and discrimination

**DOI:** 10.1186/s41235-018-0144-1

**Published:** 2018-12-13

**Authors:** Li Z. Sha, Roger W. Remington, Yuhong V. Jiang

**Affiliations:** 1 Department of Psychology, University of Minnesota, 75 East River Road, S506 Elliott Hall, Minneapolis, MN 55455 USA; 2 Center for Cognitive Sciences, University of Minnesota, Minneapolis, MN USA; 3 School of Psychology, University of Queensland, St. Lucia, QLD Australia

**Keywords:** Spatial attention, Visual search, Location probability learning, Attentional priority map, Attention training

## Abstract

The visual environment contains predictable information - “statistical regularities” - that can be used to aid perception and attentional allocation. Here we investigate the role of statistical learning in facilitating search tasks that resemble medical-image perception. Using faux X-ray images, we employed two tasks that mimicked two problems in medical-image perception: detecting a target signal that is poorly segmented from the background; and discriminating a candidate anomaly from benign signals. In the first, participants searched a heavily camouflaged target embedded in cloud-like noise. In the second, the noise opacity was reduced, but the target appeared among visually similar distractors. We tested the hypothesis that learning may be task-specific. To this end, we introduced statistical regularities by presenting the target disproportionately more frequently in one region of the space. This manipulation successfully induced incidental learning of the target’s location probability, producing faster search when the target appeared in the high-probability region. The learned attentional preference persisted through a testing phase in which the target’s location was random. Supporting the task-specificity hypothesis, when the task changed between training and testing, the learned priority did not transfer. Eye tracking showed fewer, but longer, fixations in the detection than in the discrimination task. The observation of task-specificity of statistical learning has implications for theories of spatial attention and sheds light on the design of effective training tasks.

## Significance

Misses in routine cancer screening can be surprisingly high, a problem attributed, in part, to perceptual errors and attentional limits. Here we tested whether a search task involving medical-image-like stimuli benefited from statistical learning of the target’s probable locations. Participants were assigned one of two tasks: detecting a heavily camouflaged low-contrast target in noise; and discriminating a high-contrast target from similar distractors. By placing the target frequently in one region of the image, we trained participants to prioritize the high-probability region. We found that location probability learning facilitated both tasks, but learning did not transfer when the task changed. These findings suggest that statistical learning may facilitate natural search behaviors in a task-specific manner.

## Background

Human error is a major cause of accidents, contributing to > 90% of motor vehicle crashes (National Motor Vehicle Crash Causation Survey, 2008). Human error is also surprisingly common in medical-image perception. False negatives in routine breast cancer screening are as high as 20–30% (Evans, Georgian-Smith, Tambouret, Birdwell, & Wolfe, 2013; Krupinski, 2015). These errors are attributed, in part, to a limit in visual attention. For example, conspicuous anomalies may be missed when radiologists’ attention is diverted to other aspects of the image (Wolfe, 2016). What mechanisms can be used to reduce the impact of attentional limitation? Here we examine whether training can optimize the allocation of attention so that locations of greater behavioral relevance are better attended than other locations. We also test the degree of cross-task transfer following training. Addressing these questions has implications for theories of spatial attention and may inform attentional training in applied fields, such as driving and medical-image perception.

### Spatial attention training: previous findings

Several studies on reward and statistical learning showed that training can shape spatial attention. Chelazzi et al. (2014) trained participants to associate locations with monetary reward. The training task involved visual search of geometric shapes presented in eight locations. The target was a set of triangles pointing upward and the distractors were triangles pointing downward. Finding the target yielded different amounts of reward when the target occurred in different locations. Participants learned to associate reward with location. In a subsequent testing phase, participants searched for characters among symbols that were briefly presented in the same eight locations as before. Even though the task changed and reward was no longer provided, participants were more accurate at finding targets when they occurred in the previously high-reward locations than other locations (Chelazzi et al., 2014). The cross-task transfer suggests that learning-induced changes are task-general.

Analogous results are found using statistical learning. Jiang, Swallow, Rosenbaum, and Herzig (2013) used location probability learning to modify spatial attention. Participants searched for a target, such as the letter T, among distractors. Unbeknownst to them, the target more often appeared in one quadrant than in any of the other quadrants. Although most participants could not identify the high-probability quadrant, they developed a strong spatial preference for the high-probability quadrant, producing faster response time (RT) on trials when the target was in that quadrant than in the other quadrants (see also Druker & Anderson, 2010; Geng & Behrmann, 2002; Miller, 1988). Like reward-induced changes in attention, location probability learning has enduring effects. The spatial bias toward the high-probability locations persists for several hundred trials after the target’s location becomes equi-probable. Cross-task transfer was observed in visual search tasks, such as between a T-among-L search task and a 2-among-5 search task, between a T-among-L search task and an inefficient line orientation search task, and between two versions of the T-among-L search tasks that differed in difficulty (Jiang, Swallow, Won, Cistera, & Rosenbaum, 2015). Transfer occurred even when the display changed conspicuously, such as when participants were trained with white items and tested with black items (Jiang, Swallow, et al., 2015) and when they were trained to find a T-among-L and tested to find an arrow in natural scenes (Salovich, Remington, & Jiang, 2017).

Other studies, however, challenged the idea that search history affects attention in a task-general way. Several studies failed to observe effects of monetary reward on spatial attention for participants unaware of the reward-location association, suggesting that effects of reward depend on an explicit strategy (Jiang, Sha, & Remington, 2015; Won & Leber, 2016). Even when present, the effects of implicit reward are small. Without a reliable effect, the reward learning paradigm is ill-suited for testing whether the learned priority transfers across tasks. Studies using location probability learning have found consistent implicit learning. However, cross-task transfer does not occur in every case. First, location probability learning acquired in a T-among-L search task did not transfer to a color singleton search task, and vice versa (Jiang, Swallow, et al., 2015). Second, spatial biases acquired from a T-among-L search task did not transfer to a non-search foraging task (Jiang, Swallow, et al., 2015). In this foraging task, participants saw several Ls and had to choose one of them to reveal a hidden treasure. Despite successful location probability learning in the T-among-L search task, participants did not show preferences for the previously high-probability locations in the following treasure-hunt task (see also Gwinn, Leber, & Krajbich, 2018, for minimum transfer from visual search to choice behavior). The reverse was also true: after acquiring a bias toward a quadrant frequently hiding a treasure, participants did not perform the T-among-L task faster when the target appeared in the more highly rewarded quadrant. Finally, lack of transfer was observed even when two spatial tasks were performed concurrently. In Addleman, Tao, Remington, and Jiang (2018), participants searched for a T-among-H overlaid on four natural scenes, one per quadrant. They were asked to identify the T’s orientation and to memorize all the background scenes. When the T more often appeared in one quadrant, participants became faster and more accurate at finding the T in the high-probability quadrant. However, the spatial bias did not extend to the scene task. Memory for the scene in the visual search task’s high-probability quadrant was no better than that for scenes in the low-probability quadrants (Addleman et al., 2018).

### Current study

The findings reviewed above suggest that statistical learning may facilitate visual search, implicating this mechanism in medical-image perception. In fact, some researchers suggest that tumor search relies on an initial stage of global image analysis (Kundel, Nodine, Conant, & Weinstein, 2007). Within a single glimpse, radiologists can detect the presence or absence of tumors at above-chance levels, even though they were at chance in localizing the tumor (Evans et al., 2013; Evans, Haygood, Cooper, Culpan, & Wolfe, 2016). In addition, the sort of location probability learning investigated in the laboratory may also occur in medical-image perception. The locations of tumors are constrained by anatomy. For example, the heart is a prominent structure of a chest X-ray. When scanning for lung cancer, radiologists tend to deploy attention to regions of the lung rather than the heart or the rib cage (Drew, Võ, & Wolfe, 2013). In addition, when pelvic cancer metastasizes to the brain, it has a higher concentration in the posterior fossa than other parts of the brain (Delattre, Krol, Thaler, & Posner, 1988). The presence of statistical regularities in the tumor’s locations affords an opportunity for location probability learning. However, nearly all relevant laboratory studies have used search tasks that differ significantly from applied tasks, such as tumor search in medical imaging. Unlike laboratory search tasks, tumors are difficult to segment from background tissue. This difference raises questions about the utility of location probability learning in tasks that resemble medical imaging perception.

**Fig. 1:**
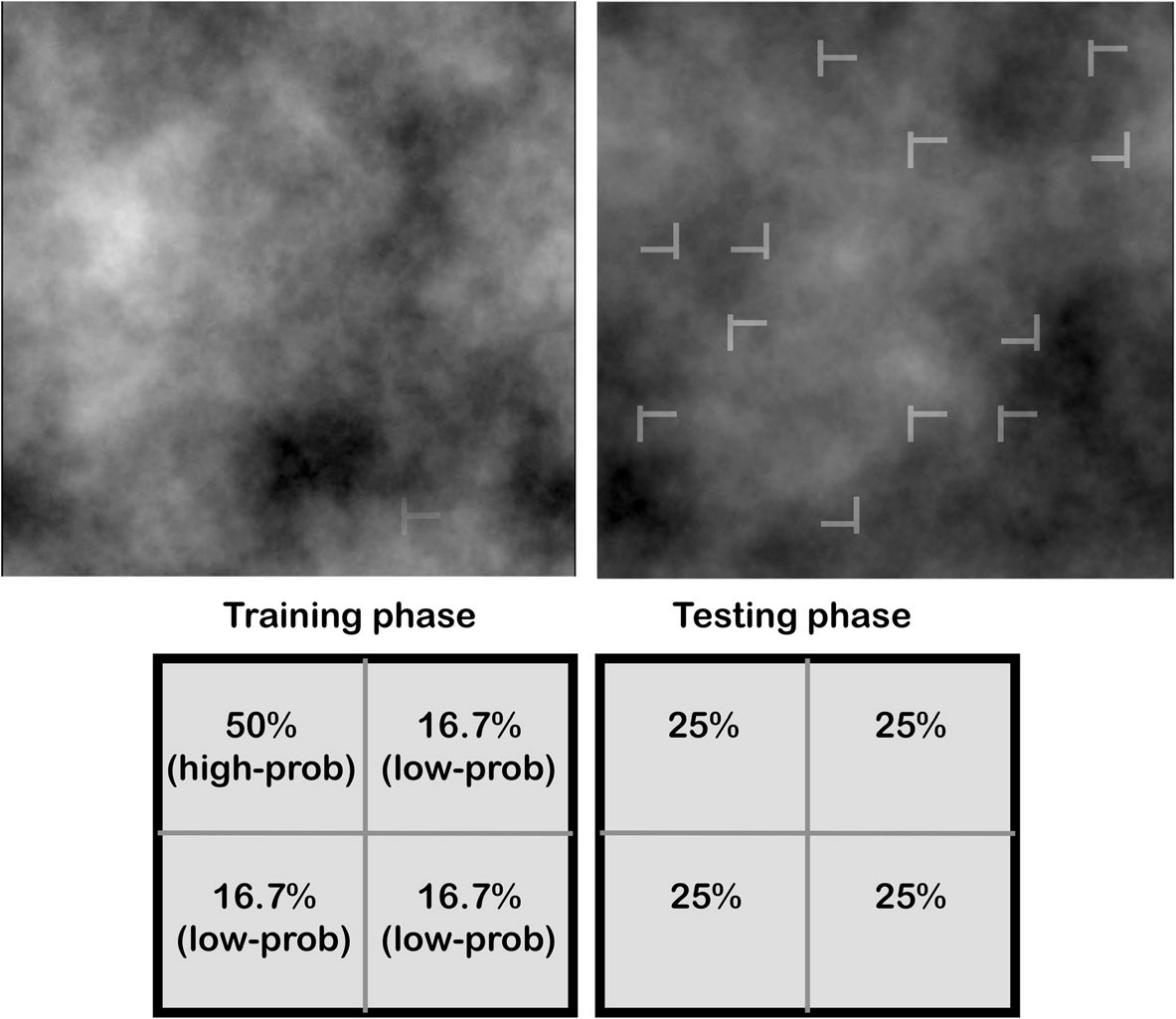
*Top*: Sample visual search trials. *Top left*: The detection task. For illustrative purposes, the noise opacity used in this example (92%) differs from that used in the actual experiment (mean 93%). *Top right*: The discrimination task. *Bottom*: The target’s location probability in the two phases of the experiment

The goal of the present study is to examine location probability learning using stimuli and tasks that are more similar to medical-image search than those used in previous studies. We employed two visual search tasks inspired by Drew, Cunningham, and Wolfe (2012) to investigate two components of medical-image search. In medical imaging, radiologists face at least two types of search problems: detecting tumors among highly confusable noise (“detection”); and differentiating abnormal from normal tissues once a candidate anomaly is demarcated (“discrimination”). The tasks used in this study, adapted from Drew et al. (2012), are an approximation to these problems. Specifically, the detection task required participants to find a low-contrast T heavily camouflaged in 1/*f*^*3*^ noise, which has similar power spectrum as mammograms (Burgess, Jacobson, & Judy, 2001). The discrimination task increased the T’s signal-to-noise ratio but presented the T among similar-looking Ls (Fig. 1). We examined location probability learning by making the target disproportionately likely to appear in one region. We asked two questions. First, can one improve search in regions that are more likely to contain a target? Second, does training in one task transfer to the other?

## Experiment 1

Studies on location probability learning have predominantly used well-segmented stimuli, typically letters (Geng & Behrmann, 2002; Jiang, Swallow, Rosenbaum, & Herzig, 2013; Miller, 1988). Experiment 1 aimed to establish location probability learning with low-contrast stimuli embedded among noise. We examined whether participants can acquire location probability learning in the detection and the discrimination tasks. We also tested the persistence of the learned spatial preference.

Participants were randomly assigned to perform either the detection or the discrimination task. In 40% of the trials, the target was absent, requiring participants to press the spacebar. In the other 60% of the trials, the target was present and was equally like to be rotated to the left or to the right. In these trials, participants reported the target’s orientation. The target’s spatial distribution was manipulated on target-present trials. In the training phase, on target-present trials, the target appeared in one quadrant 50% of the time and in each of the other three quadrants 16.7% of the time. Immediately following seven training blocks, the testing phase proceeded to probe the persistence of probability learning. In the testing phase, on target-present trials, the target appeared in all quadrants equally often (25% of the time). The task used in the training phase was maintained in the testing phase. This experiment will be contrasted with Experiment 2, which used different tasks in the training and testing phases.

### Method

#### Participants

College students were tested in this study. Their ages were in the range of 18–26 years. All had normal or corrected-to-normal visual acuity and were naive to the purpose of the study. The study was approved by the Institutional Review Board at the University of Minnesota. Each participant provided written consent before participating.

A predetermined sample size of 16 was used in each task. This is the same sample size as in previous studies on probability cuing (e.g. Jiang, Sha, & Remington, 2015). The effect size of location probability learning in previous studies was large (e.g. Cohen’s f = 1.11 in Jiang, Sha, & Remington, 2015). Minimum sample size to reach a power of 0.95 was 5.

Thirty-two participants completed Experiment 1. The participants were randomly assigned to perform either the detection (*N* = 16, 11 women and five men with a mean age of 20.9 years) or the discrimination task (*N* = 16, 14 women and two men with a mean age of 20.6 years).

#### Equipment

Participants were tested individually in a room with normal interior lighting. The experiment was coded using Psychtoolbox (Brainard, 1997; Pelli, 1997), implemented in MATLAB (2018). Stimuli were projected on a 19” CRT monitor (spatial resolution 1024 × 768 pixels), which has a vertical refresh rate of 75 Hz. Viewing distance was approximately 48 cm. Visual angles reported here were estimated from this distance.

#### Stimuli

Search items were placed in randomly selected locations in an invisible 10 × 10 matrix (33.5° × 33.5°; Fig. 1). The items were white embedded in noise with the power spectrum of 1/*f*^*3*^. The noise was chosen to resemble the power spectrum of mammograms (Burgess et al., 2001) and it changed from trial to trial. In the discrimination task, each quadrant contained three items. The target letter T was rotated 90°, either clockwise or counterclockwise, randomly determined on target-present trials. The distractor Ls in the discrimination task had a random orientation of 0°, 90°, 180°, or 270°. Each search item subtended 1.34° × 1.34°.

#### Procedure and design

Participants were tested in a short thresholding task before the main experiment. They completed a recognition test at the end.

##### The thresholding task

The purpose of this task was to choose an appropriate level of noise opacity in the detection task, or an appropriate level of target-distractor similarity in the discrimination task.

Participants tested in the detection task were tasked to find a heavily camouflaged letter T against 1/*f*^*3*^ noise (no Ls were presented in the detection task). Participants tested in the discrimination task searched for the letter T among letter Ls in noise. In both cases, the T was present on 60% of the trials and absent on 40% of the trials. To initiate each trial, participants clicked on a white fixation square (0.4° × 0.4°) placed in a random location within the central 1.5°. The mouse click required eye–hand coordination and ensured that the fixation was centered at the start of a trial. The search display then appeared and remained until participants made a response. Participants pressed the spacebar if they thought the T was absent and an arrow key to indicate the T’s orientation if they thought it was present. Correct trials were followed by three chirps; incorrect trials were followed by a low buzz, with no feedback about the position of the target. Task instructions emphasized both accuracy and RT.

The thresholding procedure of the detection task used four levels of noise opacity (91%, 92%, 93%, or 94%). The thresholding procedure of the discrimination task used four levels of target-distractor similarities. Specifically, the letter Ls had an offset at the intersection in the range of 23–26 pixels, making them increasingly dissimilar to the target (in comparison, the offset for the target T was 16 pixels). There were 15 trials of each opacity or similarity level, presented in a random order. The opacity or similarity level that yielded at least 77.8% accuracy and response times of approximately 3 s was selected for the main experiment. The mean noise opacity level used in Experiment 1 was 93% in the detection task. The mean similarity level used in the discrimination task was a 24-pixel offset.

##### Main experiment

Each participant completed 440 experimental trials, divided into 11 blocks of 40 trials each. The trial sequence was the same as in the thresholding task. The target was absent on 40% of all trials. The first seven blocks comprised training, during which the target T, when present, appeared in one (“high-probability”) quadrant 50% of the time and in each of the other three (“low-probability”) quadrants 16.7% of the time. The high-probability quadrant was counterbalanced across participants. The last four blocks comprised testing, during which the target T, when present, was equally likely to appear in any quadrant (25% of the time). Participants were not informed of the T’s location distribution.

##### Recognition test

At the completion of the experiment, we probed explicit awareness of the target’s location probability. Participants were first asked whether they thought the target was equally likely to appear anywhere on the display or whether it appeared in some locations more often than others. Regardless of their answer, they were then informed that the target appeared in some locations more often and asked to click the region where the target most often appeared. Data from the recognition task will be presented following the report of both experiments.

### Results

In the detection task, accuracy was 91.2% on target-present trials and 99.1% on target-absent trials (i.e. the false alarm rate was 0.9%). In the discrimination task, accuracy was 91.0% on target-present trials and 99.2% on target-absent trials (false alarm 0.8%). In both tasks, RT was much longer on target-absent than target-present trials (detection: 5357 ms vs 1661 ms; discrimination: 4716 ms vs 2596 ms). Because target-absent trials were uninformative of location probability learning, we examined data from target-present trials.

**Fig. 2:**
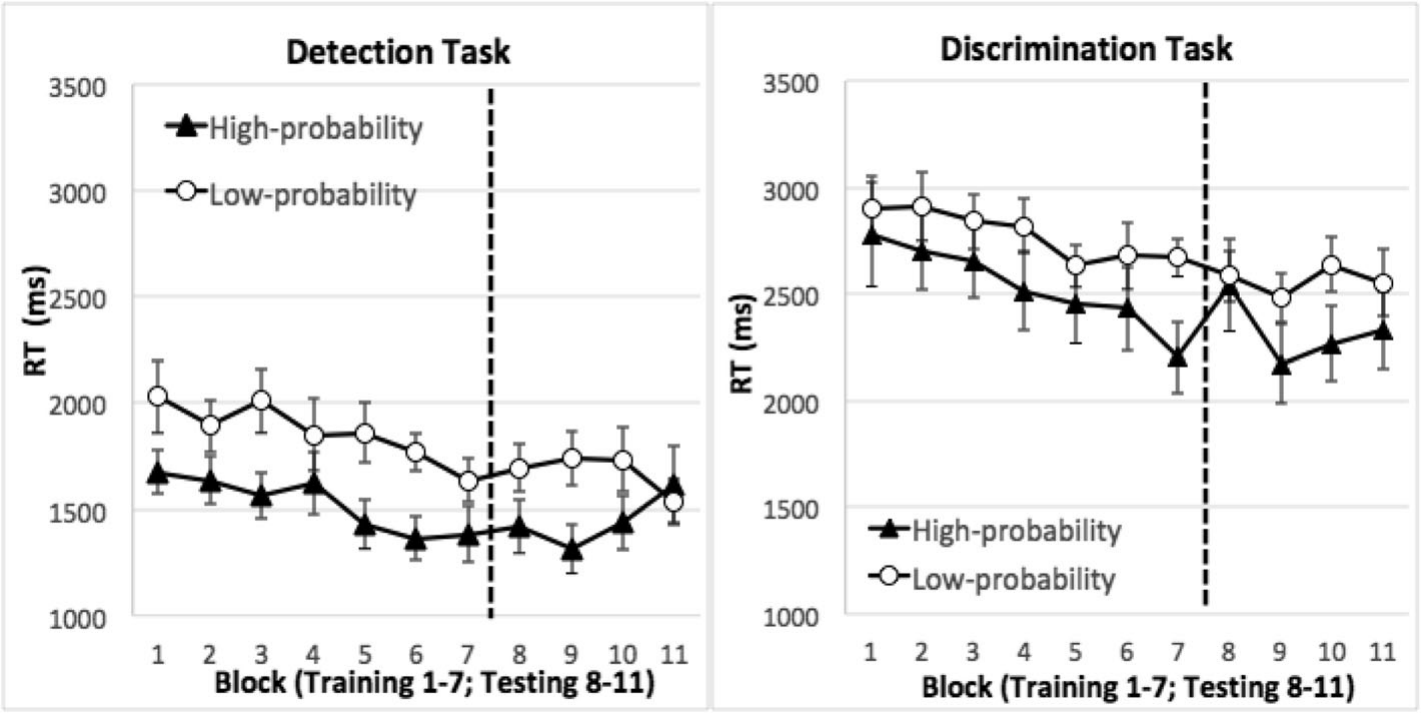
Target-present RT data from Experiment 1. The first seven blocks were the training phase (the target appeared in the high-probability quadrant disproportionately often). The last four blocks were the testing phase (the target appeared in all quadrants equally often). *Error bars* show ±1 S.E. of the mean

On target-present trials, accuracy did not differ between the high-probability quadrant and the low-probability quadrants, *t*(15) = 1.63, *p* = 0.13 in the detection task, or *t* < 1 in the discrimination task. Data analyses were conducted on RT from correct trials, excluding outliers (< 250 ms, 0% of the trials, or > 10s, 0.17% of the trials). Figure 2 displays the mean RT.

**Fig. 3:**
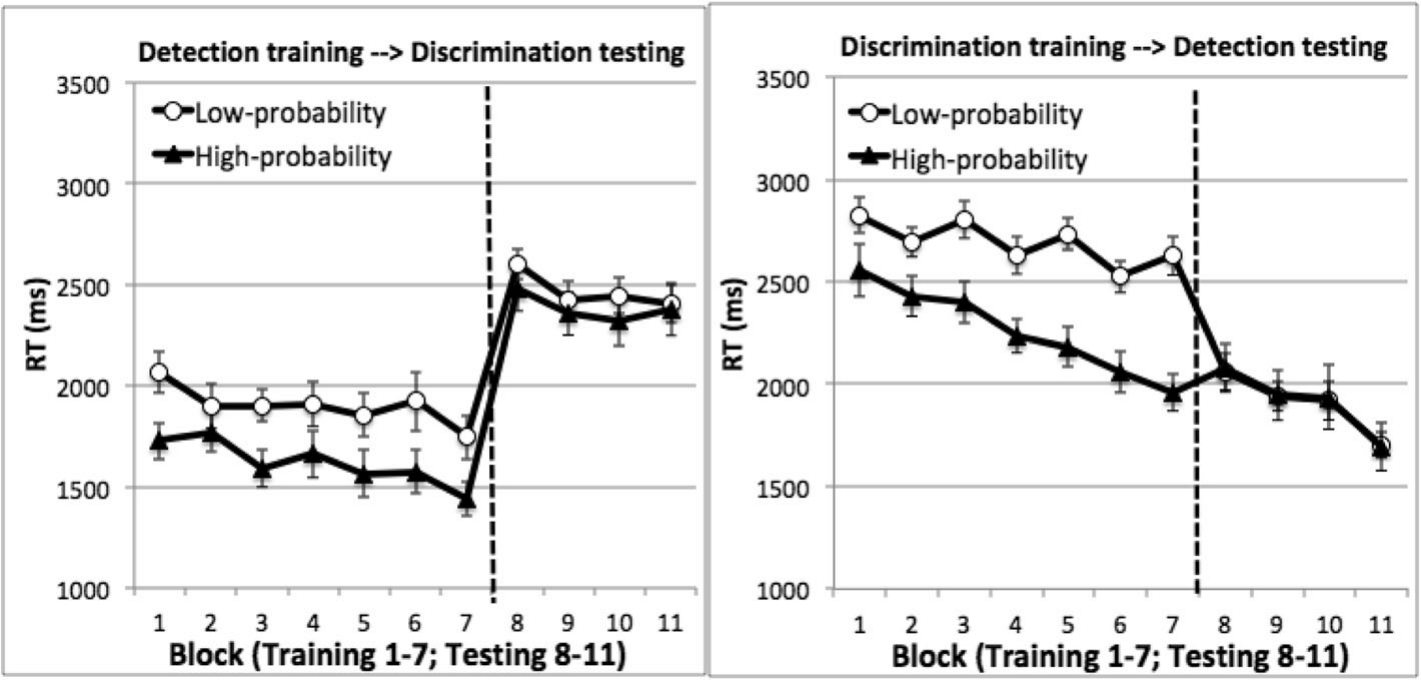
Mean RT on target-present trials of Experiment 2. *Left*: Participants were trained in the detection task and tested in the discrimination task. *Right*: Participants were trained in the discrimination task and tested in the detection task. There were seven training blocks and four testing blocks. *Error bars* show ±1 S.E. of the mean

#### Training phase

This phase revealed the acquisition of location probability learning. RT was faster when the target was in the high-probability rather than low-probability quadrants. This was verified in an ANOVA with task (detection or discrimination) as a between-subject factor, target quadrant (high versus low probability quadrants) and training block (1–7) as within-subject factors. Location probability learning was reflected in the significant main effect of the target’s quadrant, *F*(1,30) = 9.40, *p* = 0.005, *η*_*p*_^2^ = 0.24. In addition, search speed was faster in later blocks than earlier ones, producing a significant main effect of training block, *F*(6,180) = 7.20, *p* < 0.001, *η*_*p*_^2^ = 0.19. Even though we used a thresholding procedure to titrate individual participants’ search RT, RT was longer in the discrimination task than the detection task, yielding a significant main effect of task, *F*(1,30) = 52.78, *p* < 0.001, *η*_*p*_^2^ = 0.64. None of the interaction effects were significant, *F*s < 1. Thus, we successfully induced location probability learning using X-ray like stimuli.

#### Testing phase

The testing phase probed the durability of location probability learning. In this phase, the target was randomly placed and was equi-probable across all quadrants. Thus, inter-trial repetition was no more likely in the high-probability than the low-probability quadrants. Nonetheless, location probability learning persisted in the testing phase. An ANOVA using task, target’s quadrant, and testing blocks as factors revealed a significant main effect of the target’s quadrant, showing that participants were faster finding the target in the previously high-probability quadrant than the previously low-probability quadrants, *F*(1,30) = 4.90, *p* = 0.04, *η*_*p*_^2^ = 0.14. RT did not change across blocks in the testing phase, *F*(3, 90) = 1.19, *p* = 0.32 for the main effect of block. RT was faster in the detection task than in the discrimination task, *F*(1,30) = 39.84, *p* < 0.001, *η*_*p*_^2^ = 0.57 for the main effect of task. The persisting probability cuing was comparable between the detection and discrimination tasks, *F* < 1 for the interaction between target quadrant and task. Probability cuing declined marginally with prolonged testing, *F*(3, 90) = 2.44, *p* = 0.07, *η*_*p*_^2^ = 0.08 for the interaction between target quadrant and testing block. This suggests that as reinforcement of the high-probability quadrant was withdrawn, extinction of the location probability learning slowly occurred. Block did not interact with task, *F* < 1, neither was the three-way interaction significant, *F*(3, 90) = 1.69, *p* = 0.17.

### Discussion

Experiment 1 showed that participants responded to the target faster when it occurred in a high-probability region than when it occurred in a low-probability region, an effect that persisted in the testing phase. These results extended previous findings to stimuli that were heavily camouflaged in noise. To our knowledge, this is the first time that location probability learning has been found with stimuli that resemble medical images. Experiment 1 also showed that the magnitude, pace, and persistence of location probability learning were comparable between the detection and discrimination tasks, though RT in the detection task was faster than that in the discrimination task.

In both tasks, the RT advantage in the high-probability quadrant was already significant in Block 1. This could be due to inter-trial location repetition priming given that the target was more likely to repeat its quadrant in the high-probability quadrant than in the low-probability quadrants. To verify that there were no systematic differences in RT across quadrants, we examined RT on the first trial in which the target was in the high-probability quadrant and the first trial in which the target was in any of the low-probability quadrants. These data represented the “first” encounter of the conditions. We found comparable RTs between these two types of trials (2188 ms in the high-probability quadrant, 2220 ms in the low-probability quadrants, *t*(15) = 0.08, *p* = 0.94 in the detection task, 3327 ms in the high-probability quadrant, 3186 ms in the low-probability quadrants, *t*(15) = 0.35, *p* = 0.73 in the discrimination task), suggesting that the conditions were equivalent before location repetition or probability learning. Having ruled out systematic quadrant differences, the early appearance of facilitation in the high-probability quadrant could be due either to true location probability learning occurring early or inter-trial priming due to greater repetition of the approximate location of the targets for the high-probability quadrant. The sustained benefit for the high-probability quadrant in the testing phase, when targets occur equally in all quadrants, is evidence that true probability learning has occurred.

The RT advantage in the high-probability quadrant could reflect a facilitation of search efficiency; alternatively, participants could be faster in making a decision after the target had been found. However, previous studies that manipulated set size have consistently found a reduction in search slope. Search efficiency, as indexed by the slope in the linear function relating RT to set size, is greater in the high-probability than low-probability quadrants (Jiang, Swallow, & Rosenbaum, 2013; Sisk, Twedell, Koutstaal, Cooper, & Jiang, 2018). As we will show in Experiment 2, eye movement data also provide evidence that the location probability manipulation affects search shortly after the onset of the search display.

The design of our experiment included a region that was more likely to contain the target than the rest of the visual field. Conversely, location probability learning may be induced by including a region less likely to contain the target than the rest of the visual field. Although we did not use the latter design, others have varied location probability in a graded fashion (e.g. Druker & Anderson, 2010); search priority followed the gradient. In fact, even when the probability manipulation is binary, its effect is continuous across space – locations farther from the high-probability quadrant are less well attended than nearby locations (Jiang, Sha, & Sisk, 2018). Other studies have found that locations frequently containing distractors are better ignored (Wang & Theeuwes, 2018), suggesting that location probability learning can both increase target priority and reduce distractor priority.

## Experiment 2

The sensitivity of both the detection and discrimination tasks to location probability learning raises an important question about the transferrability of learning. If changes in spatial attention following training readily transfer across tasks, then the design of training tasks may be guided by convenience. For example, any stimuli and tasks might be used for training in medical imaging, as long as the spatial regularities are maintained. However, studies reviewed earlier suggest that location probability learning does not always transfer between tasks, especially if one of the two tasks does not involve visual search (e.g. treasure hunt or scene memory). Although both the detection and discrimination tasks used in this study involved visual search, differences in how well search items could be segmented may influence how people shift attention in these tasks.

Participants in Experiment 2 were randomly assigned to acquire location probability learning in either the detection or the discrimination task. Unlike Experiment 1, the task changed in the testing phase: from detection to discrimination or vice versa. We examined whether location probability learning acquired in one task transferred to the other.

We included eye tracking for a subset of the participants. This measure yielded insights into potential differences in how search was conducted. We examined whether the two tasks involved serial search (e.g. participants make multiple fixations before finding the target) and, if so, whether they differed in the number and duration of fixations. Eye tracking also provided an additional measure of a search habit: the direction of the first saccadic eye movement. Previous studies showed that location probability learning not only facilitated RT, but also increased the proportion of first saccades toward the high-probability quadrant (Jiang, Won, & Swallow, 2014; Salovich et al., 2017). Discrepancies sometimes occurred, however. The first-saccadic preference emerged more slowly than the RT advantage (Salovich et al., 2017). These findings suggest that covert attention – attentional shifts without eye movements – rely on similar, but not identical, mechanisms as overt shifts of attention with eye movements. Differences between the two raise the possibility that RT and first saccades may show different patterns of learning and cross-task transfer, a possibility tested in Experiment 2.

### Method

#### Participants

Sixty-four college students completed Experiment 2. All participants were drawn from the same participant pool. The first 32 participants were tested without an eye tracker. Among them, a random half were trained in the detection task and tested in the discrimination task, whereas the task assignment was reversed for the other half. Eye tracking was added for the last 32 participants. A random half of these participants were trained in the detection task and tested in the discrimination task and the other half were assigned the opposite tasks. Altogether, 32 participants completed the detection training (26 women and six men, mean age 20.0 years) and 32 participants completed the discrimination training (26 women and six men, mean age 20.5 years).

#### Procedure and design

Similar to Experiment 1, participants first underwent a thresholding task to determine the noise opacity for the detection task and the target-distractor similarity for the discrimination task. Thresholding was done on both tasks in separate blocks, counterbalanced in order between participants. The mean noise opacity level used in Experiment 2 was 93% in the detection task. The mean similarity level was an offset of 24 pixels.

Next, the detection training group carried out the detection task in seven blocks, then switched to the discrimination task for four blocks. The discrimination training group carried out the discrimination task in seven blocks, then switched to the detection task for four blocks. In both groups, the seven training blocks involved a biased target distribution: the target, when present, appeared in a high-probability quadrant on 50% of the trials and in each of the other quadrants 16.7% of the trials. The last four testing blocks involved an unbiased target distribution: the target, when present, appeared in each quadrant 25% of the time. This experiment was comparable in design to that of Experiment 1. The key difference is that the task changed between training and testing. Recognition test was conducted at the completion of the visual search task.

#### Eye tracking

Eye-tracking participants rested their head on a chinrest. An EyeLink 1000 eye tracker (SR research Ltd., Mississauga, ON, Canada) tracked the left eye at a sampling rate of 2000 Hz. Eye position was calibrated before the experiment and verified before each trial. Recalibration was done as needed. The eye tracker recorded the eye position and information about saccades and fixations. We focused on: (1) the number of fixations per trial; (2) the duration of each fixation; and (3) the direction of the first saccadic eye movement after trial onset.

#### Statistical analysis

In addition to repeated measures ANOVA, we performed a Bayesian analysis on the testing phase data. In the case of a null effect, the Bayesian analysis tests whether a lack of an effect is more plausible than the presence of an effect. This Bayesian analysis was implemented in the BayesFactor package in *R* (Rouder & Morey, 2012; Rouder, Morey, Speckman, & Province, 2012). We used the default prior (Cauchy prior) in this package, which has been shown to be appropriate for the vast majority of designs in experimental psychology (Rouder et al., 2012). We used a top-down model comparison to assess the evidence for or against probability cuing in the testing phase. This procedure first constructed a full model including all terms. Next, it took out each term one at a time and compared the resulting model with the full model. Each term yields a Bayes Factor, which describes the degree to which the model omitting that term is preferred over the full model. For example, a Bayes Factor of 5 implies that a model omitting the term is five times more plausible than a model including it. In other words, it is five times more likely that term does not have an effect than it does.

### Results

#### Behavioral data

Behavioral data were obtained from the whole sample. Accuracy was 98.8% on target absent trials (false alarm rate 1.2%). On target-present trials, accuracy was unaffected by the target’s quadrant. It was 89.1% in the high-probability quadrant, 88.4% in the low-probability quadrants, *t*(15) = 1.06, *p* = 0.30 for the detection-training participants; 90.9% in the high-probability quadrant, 89.2% in the low-probability quadrants, *t*(15) = 1.78, *p* = 0.09 for the discrimination-training participants. RT was longer on target-absent than target-present trials (5698 ms vs 1758 ms for the detection-training participants and 4784 vs. 2471 ms for the discrimination-training participants). We examined mean RT from correct target-present trials, excluding outliers (< 250 ms: 0.01% of the trials; > 10 s: 0.27% of the trials). Figure 3 displays these results.

The training phase was the same as in Experiment 1. Replicating Experiment 1’s finding, we found significant location probability learning. An ANOVA using task, target quadrant, and training block as factors showed a significant main effect of target quadrant, as RT was faster when the target was in the high-probability quadrant, *F*(1, 62) = 47.90, *p* < 0.001, *η*_*p*_^2^ = 0.44. RT also became faster in later blocks, producing a significant main effect of block, *F*(6, 372) = 11.65, *p* < 0.001, *η*_*p*_^2^ = 0.16. RT was faster in the detection task than the discrimination task, *F*(1, 62) = 55.94, *p* < 0.001, *η*_*p*_^2^ = 0.47. The lack of interaction between target quadrant and task suggests that probability cuing was comparable between the two tasks, *F*(1, 62) = 2.10, *p* = 0.15. Improvement in RT across training blocks was larger in the discrimination task than the detection task, *F*(6, 372) = 2.17, *p* = 0.04, *η*_*p*_^2^ = 0.03 for the interaction between block and task. None of the other interaction effects were significant, largest *F*(6, 372) = 1.79, smallest *p* = 0.10.

Even though participants acquired probability cuing, this effect did not transfer in the testing phase when the task changed. An ANOVA using task, target quadrant, and testing block as factors showed no effects of target quadrant, *F* < 1. RT improved across blocks, producing a significant main effect of testing block, *F*(3, 186) = 5.66, *p* = 0.001, *η*_*p*_^2^ = 0.08. RT was faster in the detection task than the discrimination task, *F*(1, 62) = 38.49, *p* < 0.001, *η*_*p*_^2^ = 0.38 for the main effect of task. Target quadrant did not interact with block, neither did it interact with task, *F*s < 1, and the three-way interaction was not significant, *F* < 1.

To examine the strength of the null effect in relation to the presence of a transfer effect, we conducted a Bayesian analysis on the effect of target quadrant in the testing phase (see “Method”). The Bayesian analysis provides strong evidence that location probability learning did not transfer to the testing phase when the task changed. The Bayes factor of target quadrant was 8.99, suggesting that it was nine times more likely that target quadrant did not affect RT than it did.

The above analysis combined data across all 64 participants who produced behavioral data. Note that half of these were tested on an eye tracker and the other half were not. When “eye-tracking status” was included as a between-group factor in the analysis, this factor did not interact with any of the experimental factors. In the training phase, the interaction between eye-tracking status and target’s quadrant was not significant, *F*(1, 62) = 1.16, *p* > 0.28. Location probability learning was significant in each group, *F*(1, 31) = 20.09, *p* < 0.001, *η*_*p*_^2^ = 0.39 for those with eye-tracking; *F*(1, 31) = 27.11, *p* < 0.001, *η*_*p*_^2^ = 0.47 for those without eye-tracking. In the testing phase, there was no interaction between eye-tracking status and target’s quadrant, *F*(1, 62) = 1.13, *p* > 0.29. Transfer of learning was not significant for either the eye-tracked group, *F* < 1, or those without eye tracking, *F* < 1.

#### Eye movement data: fixation pattern

##### Training phase

**Fig. 4:**
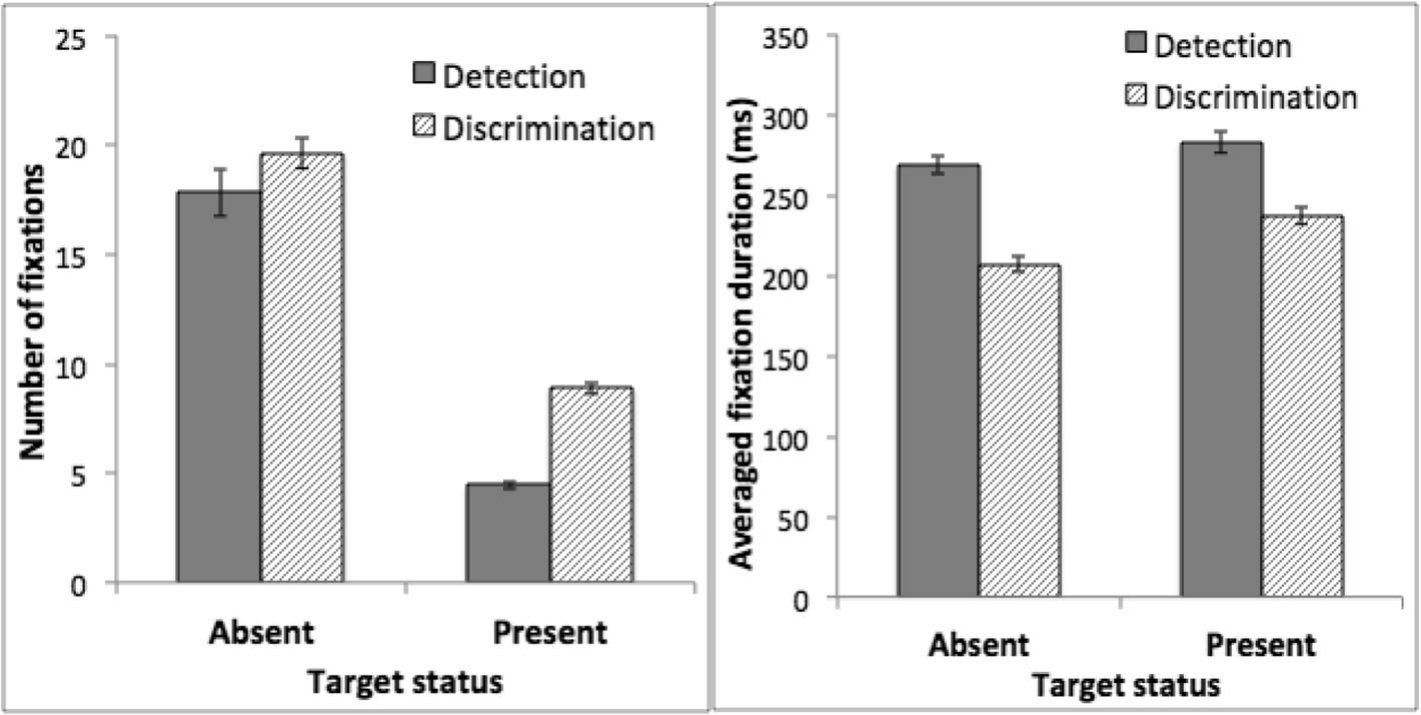
Fixation data from the training phase of Experiment 2. *Left*: Average number of fixations per trial. *Right*: Mean fixation duration. *Error bars* show ±1 S.E. of the mean

Differences in eye movement provided insight into the lack of transfer between tasks. Both tasks entailed a large number of fixations (Fig. 4, left). Participants made more fixations on target-absent trials than target-present trials. We conducted an ANOVA on the number of fixations, using task as a between-subject factor and target status (target-present or target-absent) as a within-subject factor. This analysis showed a significant main effect of target status, with more fixations on target-absent trials, *F*(1, 30) = 422.87, *p* < 0.001, *η*_*p*_^2^ = 0.93. Participants performing the discrimination task made more fixations than those performing the detection task, *F*(1, 30) = 18.06, *p* < 0.001, *η*_*p*_^2^ = 0.38, a difference that was larger on target-present trials than target-absent trials, *F*(1, 31) = 5.08, *p* = 0.03, *η*_*p*_^2^ = 0.15 for the interaction between task and target status.

Although participants in the discrimination training task made more fixations, on average each fixation was briefer (Fig. 4, right). An ANOVA using target status as a within-subject factor and task as a between-subject factor showed that fixation duration was shorter on target-absent trials than target-present trials, *F*(1, 30) = 40.74, *p* < 0.001, *η*_*p*_^2^ = 0.58. Fixation duration was longer in the detection task than in the discrimination task, *F*(1, 30) = 55.34, *p* < 0.001, *η*_*p*_^2^ = 0.65, a difference that was larger on target-absent than target-present trials, *F*(1, 30) = 5.40, *p* = 0.03, *η*_*p*_^2^ = 0.15 for the interaction between task and target status.

##### Testing phase

**Fig. 5:**
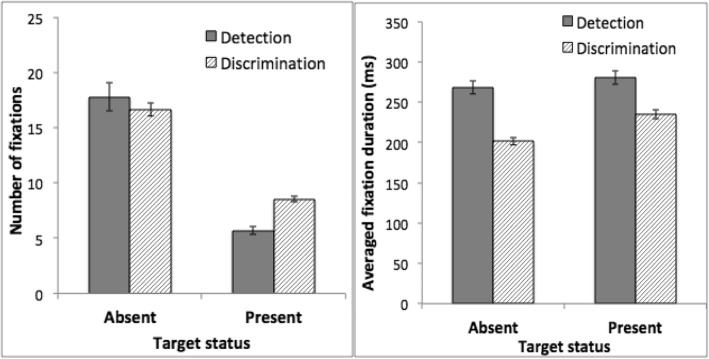
Fixation data from the testing phase of Experiment 2. *Left*: Average number of fixations per trial. *Right*: Mean fixation duration. *Error bars* show ±1 S.E. of the mean

The pattern of fixation data was replicated in the testing phase (Fig. 5). Specifically, participants performing the discrimination task made more fixations than those performing the detection task, particularly on target-present trials, *t*(30) = 6.59, *p* < 0.001. Mean fixation duration was briefer in the discrimination task than the detection task, *F*(1, 30) = 36.86, *p* < 0.001, *η*_*p*_^2^ = 0.55. Other aspects of the statistical analyses were similar to those of the training phase and the details are omitted.

The fixation data showed that both detection and discrimination tasks involved a large number of fixations, supporting the assumption that the tasks required serial search. Differences between the two tasks were also apparent. Participants made more fixations in the discrimination task than in the detection task. However, each fixation was briefer in the discrimination task.

#### Eye movement data: first-saccadic eye movements

**Fig. 6:**
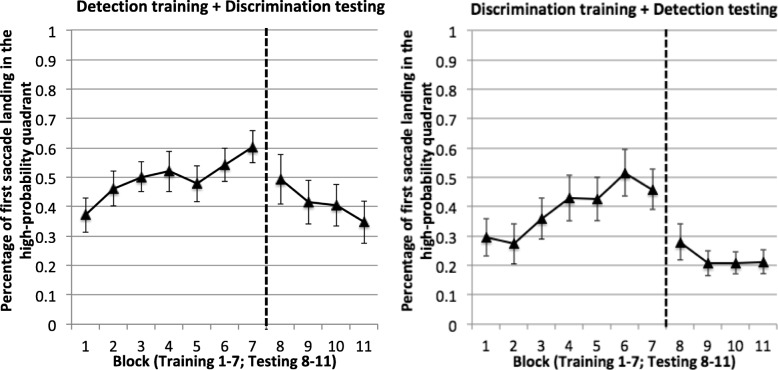
Direction of the first saccadic eye movement in Experiment 2. Plotted are the percentage of trials where the first saccadic eye movement was directed toward the high-probability quadrant on target-absent trials. Higher values indicate a stronger saccadic bias toward the high-probability quadrant. *Error bars* show ±1 S.E. of the mean

Not only were people faster in finding the target in the high-probability quadrant, but they also acquired a tendency of saccading toward that quadrant first. This effect was most clearly revealed on target-absent trials, where saccade could not have been influenced by the presence of target features (Fig. 6). With four quadrants, the chance rate of saccading toward the high-probability quadrant is 25%. In the training phase (Blocks 1–7), the mean percentage of trials with first saccades to the high-probability quadrant was 49.8% in the detection task and 39.6% in the discrimination task, both of which were significantly higher than 25%, *t*(15) = 4.97, *p* < 0.001 for the detection task and *t*(15) = 2.28, *p* = 0.04 for the discrimination task. An ANOVA using task and training blocks as factors showed that participants’ tendency to saccade toward the high-probability quadrant increased across training blocks, *F*(6, 180) = 9.36, *p* < 0.001, *η*_*p*_^2^ = 0.24 for the main effect of block. The main effect of task (*F*(1, 30) = 1.60, *p* = 0.21) and the interaction between block and task (*F*(6, 180) = 1.37, *p* = 0.23) were not significant.

As the task changed in the testing phase, the saccade pattern also changed. Those trained in the discrimination task and tested in the detection task no longer persisted in their overt search pattern. In these participants, the mean percentage of trials with first saccades to the previously high-probability quadrant in the testing phase was 22.6%, a level not significantly higher than chance, *t* < 1. Those trained in the detection task and tested in the discrimination task, however, showed a persisting but declining trend of saccading toward the high-probability quadrant. For these participants, the percentage of first saccades directed toward the high-probability quadrant in the testing phase – 41.5% – was significantly higher than chance, *t*(15) = 2.36, *p* = 0.03. This effect declined across testing blocks, *F*(3, 90) = 4.31, *p* = 0.007, *η*_*p*_^2^ = 0.13.

**Fig. 7:**
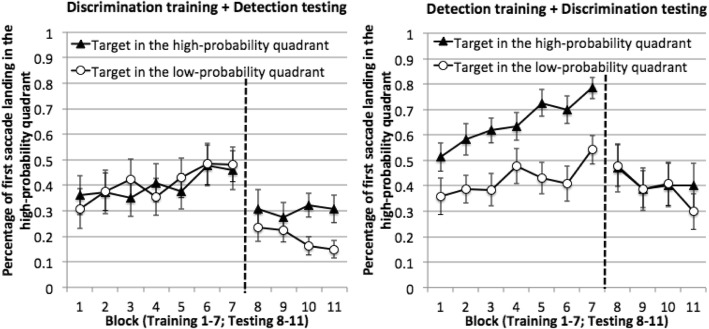
Direction of the first saccadic eye movement on target-present trials in Experiment 2. Y-axis plots the proportion of trials in which the first saccade was directed toward the high-probability quadrant. Trials were separated depending on where the target was. *Left*: Participants were trained in the discrimination task and tested in the detection task. *Right*: Participants were trained in the detection task and tested in the discrimination task. *Error bars* show ±1 S.E. of the mean

Qualitatively similar results were observed on target-present trials (Fig. 7 in the Appendix). These trials presented some complications given that the first saccades may be made after detecting target features and therefore would be influenced by where the target was on a trial. Nonetheless, the pattern of data was similar to target-absent trials. Specifically, when trained with the discrimination task, participants gradually acquired a tendency to saccade toward the high-probability quadrant first. This preference ceased when the task changed to detection. When trained with the detection task, participants also acquired a tendency to saccade toward the high-probability quadrant first. This preference was substantially reduced, though somewhat persistent, when the task changed to discrimination. Detailed results and statistical analyses that took into account the target’s location can be found in the Appendix.

Figure 7 in the Appendix showed different saccade patterns between the detection and discrimination tasks. Regardless of which phase these tasks were performed in, first saccades in the discrimination task were insensitive to the target’s actual location. The proportion of first saccades toward the high-probability quadrant was no stronger when the target itself was in the high-probability quadrant than when it was elsewhere. This suggests that the first saccades were executed before acquiring target features. In contrast, in the detection task, the proportion of first saccades toward the high-probability quadrant was stronger when the target was in that quadrant than when it was elsewhere. This suggests that first saccades were initiated after participants had analyzed the image and had some information about where the target was. In fact, first saccade latency was longer in the detection task than in the discrimination task, both in the training phase (target-absent trial means: 240 ms vs 156 ms, *t*(30) = 4.67, *p* < 0.001 on trials) and in the testing phase (226 ms vs 148 ms, *t*(30) = 5.50, *p* < 0.001).

### Discussion

Experiment 2 successfully induced a change in spatial attention in the training phase in both the detection and discrimination tasks. However, no transfer in RT was observed when the task changed. This was the case even though the two tasks were performed in the same general space, the task set was similar, and the displays had similar visual characteristics including the use of 1/*f*^*3*^ noise. On its own, the lack of transfer may be explained by differences between the two tasks. For example, the discrimination task took longer. However, differences in search RT did not prevent transfer in previous studies. Jiang, Swallow, et al. (2015) observed transfer between two T-among-L search tasks of different difficulty. The easy task had a mean RT around 1 s and the difficult task 3 s. The discrepancy in task difficulty in that case was greater than in the current study, where RT differed by about 0.5 s. A difference in display appearance (e.g. noise opacity level) also could not explain the results. Salovich et al. (2017) showed transfer between two visually very different tasks – finding a T-among-L and finding an arrow in natural scenes.

What might account for the lack of transfer in the current study? We suggest that the lack of transfer may reflect differences in how search was conducted between the two tasks. The discrimination task requires participants to make serial shifts of attention among items that are easily segmented from the background. The detection task has few candidate regions to inspect but requires longer scrutiny when one is identified. This differs from previous studies where all tasks involve serial scanning among segmented objects. The eye data supported this suggestion. The discrimination task involved a higher number of fixations than the detection task, but each fixation was briefer. In addition, the detection, but not the discrimination, task involved an initial stage of image analysis before the first saccade was made. These data suggest that the search procedures differed between the two tasks.

The target’s location probability not only enhanced search RT, but also induced a tendency to direct the first saccade toward the high-probability quadrant. Consistent with RT, the first-saccade bias acquired in the discrimination task did not transfer to the detection task. However, the saccade preference acquired in the detection task only gradually declined when the task changed to discrimination. This latter finding was not accompanied by an RT advantage. This discrepancy suggests that a habit involving saccades is harder to correct than the covert search habit indexed by RT. The lack of an RT advantage suggests that information gathered from the preferential saccades is discounted at a later level; hence, there was no RT advantage even though eye movements showed a residual preference toward the previously high-probability quadrant.

## Awareness

In the recognition test, some participants reported noticing that the target was more likely to occur in some locations than others and went on to correctly select the high-probability quadrant. These were the “aware” participants. Combining data from both experiments, there were 22 aware participants from detection-training (out of 48 total) and eight from discrimination training (out of 48). The lower level of awareness in the discrimination task may be attributed to the more demanding nature of the task owing to the presence of distractors. The remaining 66 participants were “unaware.” To examine the association between explicit recognition and location probability learning, we performed a mixed ANOVA on the training phase data from each task, using target quadrant and block as within-subject factors and awareness as a between-subject factor. In participants undergoing detection training, location probability learning was significant, *F*(1, 46) = 44.44, *p* < 0.001, *η*_*p*_^2^ = 0.49, an effect that did not interact with awareness status, *F* < 1. Similarly, in participants undergoing discrimination training, location probability learning was significant, *F*(1, 46) = 14.13, *p* < 0.001, *η*_*p*_^2^ = 0.24, but this did not interact with awareness status, *F* < 1.

To ensure that location probability learning occurred independently of awareness, we performed an ANOVA on the training phase data for unaware participants only, separately for the two tasks, using target quadrant and block as within-subject factors. Unaware participants undergoing detection training showed location probability learning (main effect of target quadrant), *F*(1, 25) = 30.68, *p* < 0.001, *η*_*p*_^2^ = 0.55. Similarly, unaware participants undergoing discrimination training showed significant location probability learning, *F*(1, 39) = 13.60, *p* < 0.001, *η*_*p*_^2^ = 0.26. Consistent with prior work (Geng & Behrmann, 2002; Jiang et al., 2018), location probability learning did not depend on explicit awareness.

Finally, to examine whether explicit awareness affected the first-saccade direction, we compared the percentage of first saccades that landed in the high-probability quadrants between the aware and unaware participants. Independent t-tests did not show significant differences between the two groups in either the training phase, *t* < 1, or the testing phase, *t*(30) = 1.13, *p* > 0.26.

## General discussion

In two experiments we investigated the efficacy of incidental probability learning to induce changes of spatial attention in faux X-ray images, with the goal of determining if patterns of attentional bias acquired through learning could be leveraged to facilitate performance in search tasks that resemble radiological image perception. The detection task required participants to segment a target from background noise. We showed that this task was sensitive to the target’s location probability. Participants preferentially searched the region where the target was frequently found. This preference was acquired incidentally. Once acquired, it persisted after the target’s location was random and equi-probable. Similar results were observed in the discrimination task, which used well-segmented elements and required a more traditional approach of discriminating the target from distractors based on shape (Wolfe, 1998). Although the faux X-ray images used here are not real, they are an intermediate stimulus between typical laboratory stimuli and radiological images. Our data suggest that location probability learning can facilitate performance in search tasks that resemble components of medical-image perception and that learning is task-specific.

Other studies, such as Evans et al. (2013, 2016), showed that radiologists relied on global image statistics to detect abnormalities in mammograms and cervical cancer images. Within a single glance, radiologists could determine the presence or absence of cancers at above-chance levels, even though they could not localize the cancer. In fact, radiologists gave higher abnormal ratings to images of apparently normal breasts that subsequently turned cancerous (Brennan et al., 2018). These findings suggest that rapid extraction of global image statistics could aid perception. The current study supports the idea that statistical regularities in medical-image-like stimuli can facilitate visual search. We identified one specific source of regularities: the location probability of the target object. This type of learning may be useful in medical image perception because tumor locations are not random (Delattre et al., 1988; Drew et al., 2013).

An important finding from the current study is that learning-induced changes in spatial attention did not transfer between the discrimination and the detection tasks. This finding extends previous findings on the task-specificity of location probability learning. Those previous studies observed a lack of transfer between search and non-search tasks, such as between a T-among-L search task and scene memory (Addleman et al., 2018) and between visual search and treasure hunt (Jiang, Swallow, et al., 2015). However, previous studies did find transfer of probability cuing between visual search tasks, including tasks that differed in RT and search efficiency or tasks that used completely different stimuli. The lack of transfer between two visual search tasks is a unique finding. This result cannot be explained by differences in search RT or display appearance, as those factors did not hinder task transfer in previous studies. Our study provides an important boundary condition for transfer of location probability learning: even tasks that both require serial search may not show transfer.

An important difference between the current study and previous work is the selection of tasks. Nearly all previous studies on location probability learning used well-segmented items. Those tasks, regardless of difficulty or visual appearance, all required discriminating well-segmented items. In the current study, however, the detection task had no clearly demarcated items. Participants made global image analysis before fixating on a candidate region. In contrast, the discrimination task showed no evidence of pre-fixation analysis. The first saccade was equally likely to land in the high-probability quadrant regardless of whether the target was actually there. The two tasks also differed in the number of fixations and their duration, suggesting that search involved different procedures in these tasks.

The task-specificity of location probability learning has implications for theories of spatial attention. Existing studies use the analogy of “maps” to describe attentional priority. Much like a real map, depictions of the priority map illustrate a static image, with hotspots in some places and cooler regions in others. This entirely spatial, or “where,” analogy of attentional priority does not readily explain why such a map should be specific to tasks. An alternative to the concept of priority map is the idea that spatial attention includes a procedural component, akin to learning oculomotor search paths. Learning to attend involves not just learning where targets are likely to be, but also how to optimize the vector of scan path to find the target (Jiang, 2017; Jiang, Swallow, Rosenbaum, & Herzig, 2013). Learning may influence attention not just by changing the weights assigned in a Cartesian coordinate space. It may also affect the preferred direction of attentional shift, the “how” of spatial orienting. Tasks that differ in search procedure may not show transfer of learning because they do not share the procedural component of search.

Location probability learning affects not only search RT, but also overt allocation of attention as indexed by eye movements. Participants directed a disproportionately high number of first saccades toward the high-probability quadrant. This tendency strengthened with training. When the task changed in the testing phase, the first-saccade bias did not carry over when participants were trained in the discrimination task but tested in the detection task. On the other hand, the first-saccade bias acquired in the detection task did carry over to the discrimination task. This bias gradually dissipated in the testing phase. This asymmetry may be explained by how the two tasks were performed. Because first saccades in the detection task were made after an initial analysis about the image, guidance from target features could override the saccade habit acquired from the discrimination task. In contrast, first saccades in the discrimination task were mainly driven by previous experience. This allowed the habit acquired from the detection task to more easily influence saccades. Regardless of the explanation, the latter pattern of data presents an intriguing contrast to the lack of a corresponding RT advantage.

Two issues relate to this finding. First, though highly correlated, eye movement and covert shift of attention reflect somewhat different mechanisms (Posner, 1980; Remington, 1980; Wu & Remington, 2003). In location probability learning, it takes about twice as long for participants to acquire the first-saccade bias as the RT advantage (Salovich et al., 2017). In addition, frequently moving one’s eyes to a quadrant is neither necessary nor sufficient for location probability learning. The RT effect was robust when participants were not allowed to move their eyes or when the display was presented too briefly for eye movements (Addleman et al., 2018; Geng & Behrmann, 2005). Conversely, frequently moving one’s eyes to a quadrant did not induce location probability learning if a goal-directed cue (e.g. a central arrow) was the source of the eye movement (Jiang, Swallow, & Rosenbaum, 2013). These findings suggest that the mechanisms supporting the first-saccade bias and covert probability cuing are partially dissociable.

Second, our study showed that even when the eyes continued to favor one quadrant, this act by itself did not convert into an RT gain. Although this finding seems surprising, it is consistent with theories of attention that embrace its multifaceted nature. Serences and Kastner (2014) summarized several mechanisms by which selective attention may influence processing. These include enhancement of the attended signal, inhibition of unattended signals, and selective readout of information from the attended channel. In other words, attention affects both perceptual and decisional processes. A saccadic bias may enhance sensory processing from the previously high-probability quadrant, but its impact will be minimal if the decision, or “readout,” process does not favor input from that region.

Our study shows that learning of statistical regularities, such as the probable locations of targets, can facilitate search. Part of the learning for medical students and residents may be to acquire the right “prior” of likely tumor locations in certain types of cancer (Delattre et al., 1988; Drew et al., 2013). Because this learning is incidental, and its persistence is not under complete conscious control, probability learning of this type can facilitate search among images that share the same probability distribution. However, such learning may have its drawbacks as well. It may hinder search when the tumor appears in low-probability regions. The latter may contribute to inattentional blindness and misdiagnosis (e.g. missing a tumor) in low-probability regions. Developing specialties within radiological training and adopting computer technologies that preferentially scrutinize low-probability regions, may help alleviate this problem.

Because changes in spatial attention are task-specific, our study suggests that statistical learning is more effective if performed on the same type of images that people are likely to encounter. This recommendation differs from most Brain Training technologies, which tout far transfer from training in specific laboratory tasks (e.g. useful field of view) to real-world tasks (such as driving). Ultimately, transfer of learning rests on shared processes between the trained task and the testing task. Our study provides an example in which transfer fails even between two search tasks performed in the same general space. It highlights the specificity of human skill acquisition, an idea that resonates with findings in other domains such as chess expertise (Bilalić, McLeod, & Gobet, 2009).

Our study is only the first step toward understanding effects of spatial training on applied tasks such as medical imaging. The search stimuli used in our study differ from tumors and other biological tissues; the prevalence of targets is much higher than the rates of tumor in medical images; the testing equipment (e.g. CRT monitor) differs from what radiologists use; and participants had no prior experience in search from images that resemble medical images. Future studies are needed to both increase the realism of the task and to employ spatial regularities informed by the distribution of tumors. One approach to increase realism is to use targets that resemble actual tumors in shape, size, texture, and contrast. Another approach is to extract the spatial distribution of tumors from various types of cancer and implement this distribution in the location probability manipulation. In addition, future study can employ tasks requiring both detection and discrimination (e.g. adding noise to the current discrimination task). Finally, collecting data on medical students and residents in training can help understand the type of spatial biases they do acquire.

## Conclusion

Using stimuli embedded in noise, this study demonstrates that statistical learning can facilitate visual search of images resembling X-rays. We showed that consistently finding a target in one region biases people toward searching that region. This effect does not transfer between a detection task, in which the target signal is heavily camouflaged, and a discrimination task involving well-segmented items. Future studies should extend the realism of these findings by characterizing the type of statistical regularities present in medical images. In addition, it will be important to elucidate the pros and cons of acquiring location probability learning in radiology and related fields.

## Appendix

Figure 7 (left) displays the first saccades of participants who were trained in the discrimination task and tested in the detection task. We separated trials in which the target itself was in the high-probability quadrant, from trials when it was in the other quadrants. In the training phase using the discrimination task, participants had a tendency to saccade toward the high-probability quadrant. This was the case both when the target itself was in the high-probability quadrant, *t*(15) = 2.40, *p* = 0.03, and when the target was in a low-probability quadrant, and *t*(15) = 2.48, *p* = 0.03. An ANOVA with block and target’s location (high-probability and low-probability) as within-subject factors showed a main effect of block, suggesting that people were increasingly more likely to saccade toward the high-probability quadrant in later blocks, *F*(6, 90) = 2.16, *p* = 0.055, *η*_*p*_^2^ = 0.13. This tendency, however, was unaffected by where the target was, *F* < 1 for the main effect of target’s location. As soon as the task changed in the testing phase, the first-saccade bias ceased. The percentage of first saccades landing in the previously high-probability quadrant did not differ from chance, both when the target occurred in the previously high-probability quadrant, *t*(15) = 1.25, *p* = 0.23, and when the target occurred in the other quadrants, *t*(15) = 1.57, *p* = 0.13. The detection task, however, was sensitive to where the target itself was. The first saccade was more likely directed to the high-probability quadrant if the target itself appeared in that quadrant rather than elsewhere, *F*(1, 15) = 13.92, *p* = 0.002, *η*_*p*_^2^ = 0.48. The main effect of block and the interaction between target’s location and block were not significant, *F*s < 1.

Results were largely similar for participants trained in the detection task and tested in the discrimination task. In the training phase, the first saccades were biased toward the high-probability quadrant both when the target itself appeared in the high-probability quadrant, *t*(15) = 9.97, *p* < 0.001, and when it appeared in a low-probability quadrant, *t*(15) = 3.35, *p* = 0.004. An ANOVA with block and target’s location as factors showed that this bias increased across training blocks, *F*(6, 90) = 6.18, *p* < 0.001, *η*_*p*_^2^ = 0.29 for the main effect of block. This bias was stronger when the target was in the high-probability quadrant than when it was elsewhere, *F*(1, 15) = 40.11, *p* < 0.001, *η*_*p*_^2^ = 0.73. Thus, first saccades in the detection task were made after participants had processed the global image statistics; hence, the saccade direction was biased toward where the target was. When the task changed in the testing phase, the first-saccade bias was retained, both when the target itself appeared in the high-probability quadrant, *t*(15) = 2.26, *p* = 0.04 and when the target appeared elsewhere, *t*(15) = 1.97, *p* = 0.067. An ANOVA using target’s location and block as factors showed that this bias marginally diminished in the testing phase, *F*(3, 45) = 2.40, *p* = 0.08, *η*_*p*_^2^ = 0.14 for the main effect of block.
